# Effects of optineurin siRNA on apoptotic genes and apoptosis in RGC-5 cells

**Published:** 2011-12-17

**Authors:** Hongyang Li, Xiuqin Ao, Juan Jia, Qingzhong Wang, Zhongzhi Zhang

**Affiliations:** 1 China Medical University, Shen Yang, Liaoning province, China; 2 CHUANG YI Biology Research Center, Shang Hai, China

## Abstract

**Purpose:**

Optineurin is a pathogenic gene associated with primary open angle glaucoma (POAG), in which the retinal ganglion cells (RGCs) are targeted. However, the functions of optineurin, particularly in RGCs, are currently not clear. We introduced optineurin siRNA into cultured retinal ganglion cell 5 (RGC-5) and PC12 cells to determine the cellular and molecular mechanisms underlying the role of optineurin in POAG.

**Methods:**

We constructed four optineurin siRNA–expressing plasmids, and transiently transfected them into PC12 cells. Quantitative real-time PCR, western blot, and fluorescent microscopy were used to determine optineurin expression and select the most effective optineurin siRNA to construct RGC-5 and PC12 stable transfected cells. Dimethylthiazolyl diphenyl tetrazolium bromide (MTT) assay and flow cytometry were applied to investigate the role of optineurin siRNA in cell growth and apoptosis. Gene microarray and quantitative real-time PCR were used to screen and validate differentially expressed genes in optineurin siRNA transfected PC12 and RGC-5 cells.

**Results:**

siRNA effectively downregulated optineurin expression in RGC-5 and PC12 stable transfected cells. Optineurin siRNA significantly inhibited cell growth and increased apoptosis in RGC-5 and PC12 cells. Microarray analysis identified 112 differentially expressed genes in optineurin siRNA transfected RGC-5 cells. Quantitative real-time PCR and western blot confirmed that the expression of brain-derived neurotrophic factor (*Bdnf*), neurotrophin-3(*Ntf3*), synaptosomal-associated protein 25(*Snap25*), and neurofilament, light polypeptide(*Nefl*) was significantly downregulated in RGC-5 and PC12 cells transfected with optineurin siRNA.

**Conclusions:**

Our study suggested that optineurin downregulation by siRNA in RGCs was an in vitro model for studying the mechanisms of optineurin effects on POAG. Neuroprotective factor and axonal transport genes may be involved in the development of POAG and could be novel targets for treating POAG due to optineurin mutation.

## Introduction

Glaucoma is the leading cause of irreversible blindness worldwide [1-3], and most of the cases are primary open angle glaucoma (POAG) [1], which is characterized by optic disc cupping and irreversible loss of retinal ganglion cells [2,3]. However, the pathogenic mechanism of POAG is not clear.

Genetic changes play an important role in the pathogenesis of glaucoma [4]. With the development of molecular genetics, in 2002 a new gene, designated as optineurin [5] (optic neuropathy inducing protein), was identified as being associated with POAG. However, the gene’s function is unclear. It has been demonstrated that optineurin binds to myosin VI in the Golgi complex and plays a crucial role in Golgi ribbon formation and exocytosis [6]. There are still arguments regarding whether optineurin inhibits or promotes apoptosis. Zhu et al. [7] found that optineurin protects cells by maintaining activation of nuclear factor-kappaB (NF-κB) activation induced by tumor necrosis factor (TNF)-alpha. However, optineurin overexpression inhibited the protective effects of E3–14.7K on TNF-alpha receptor 1-induced cell death. Recently, a study revealed that optineurin interacted with metabotropic glutamate receptors (mGluRs) and played an important role in antagonizing agonist-stimulated mGluR1a signaling [8]. Weisschuh et al. [9] used RNA interference to silence optineurin in HeLa cells, and, using microarray technology, found a series of differentially expressed genes.

Although retinal ganglion cells (RGCs) are the target cells of glaucoma, few research regarding the impact of optineurin on RGCs have been conducted. Therefore, in the present study, we used RNA interference technology to downregulate the expression of optineurin in PC12 and RGC-5 cells, a pathologic condition mimicking the POAG caused by optineurin mutation. Dimethylthiazolyl diphenyl tetrazolium bromide (MTT) assay and flow cytometry were applied to determine the effects of optineurin on proliferation and apoptosis in RGC-5 cells. To study the underlying mechanisms, we screened differentially expressed genes with gene microarray technology and validated them with quantitative real-time PCR and western blot. Our findings will help us learn the functions of optineurin. They might be also useful for treating POAG due to optineurin mutation.

## Methods

### Cell culture

PC12 and RGC-5 cell lines (ATCC) were maintained in Dulbecco’s Medium Eagle’s medium (DMEM; nvitrogen Gibco, Carlsbad, CA) supplemented with 10% fetal bovine serum, 100 μg/ml penicillin, and 100 μg/ml streptomycin. Routine testing confirmed that the cells were free of mycoplasma and viral contaminants during the entire study period.

### Construction of optineurin siRNAs and screening by transient transfection

We designed four siRNA targeting sequences according to the rat optineurin reference gene sequence (GenBank NM_145081.3) by the siRNA Target Finder Program (Silencer® Pre-designed siRNA, Ambion, Foster City, CA). BLAST was performed with the selected siRNA sequences against expressed sequence tag libraries to ensure that only a single gene (optineurin) was targeted. One scrambled siRNA (Optineurin–NC) was used as a negative control. The sequences are described in Table 1. Purified fragments were digested with BamHI/BglII and inserted into the pGPU6/GFP/Neo vector (GenPharma, Placentia, CA). All constructs were identified by sequencing. The resultant plasmids containing siRNA 1, 2, 3, and 4 and the negative control sequences were sihoptineurin-1, sihoptineurin-2, sihoptineurin-3, sihoptineurin-4, and sihoptineurinNC, respectively.

**Table 1:** Optineurin-NC sequences.

**Gene**	**Sequence (5′-3’)**
Optineurin-1 (sihoptineurin 1)	GCTGCTGCAGCAAATGAAA
Optineurin-2 (sihoptineurin 2)	GGAACTGACTGTGAGCCAAC
Optineurin-3 (sihoptineurin 3)	GGGCGATTTGAGGAGCTTTCT
Optineurin-4 (sihoptineuri N4)	GCTCCTTCAGGAACACAATAA
Optineurin-NC	UAUCGGAACCCUAGGUUCCTT (Forward)
	GGAACCUAGGGUUCCGAUATT (reverse)

Before transfection, cells were seeded into six well plates at 80% confluency for 12 h. Cell transfection was performed with Lipofectamine 2000 (Invitrogen, Carlsbad, CA) according to the manufacturer's instructions: 4.0 µg plasmids and10 µl Lipofectamine 2000 were used in each well.

To efficiently knock down optineurin, cells were transfected twice with siRNA on days 1 and 3. Quantitative real-time PCR was used to select the most effective siRNA (sihoptineurin-3) from the four candidates, which was used to establish RGC-5 and PC12 stable transfected cells.

### Stable transfection of siRNA

Cells were cultured in six well plates. Plasmid sihoptineurin-3 and plasmid sihoptineurinNC were transfected with Lipofectamine 2000 as described above. Plasmid sihoptineurinNC was used as a negative control. The cells were screened by G418 for 4 weeks, and several colonies were obtained. The same concentration of G418 was used to continue screening for another 4 weeks to obtain a positive monocolony. Survival colonies were isolated and expanded. We examined the expression of optineurin in the stable cell lines with fluorescence microscopy and western blot. We successfully established optineurin siRNA stable expression cell lines in PC12 and RGC-5 cells. We also used electron microscopy to observe the changes in the stable transfected cells.

### Transmission electron microscopy

Cells were first fixed in 2.5% glutaraldehyde for 1–4 h at 4 °C, and then fixed in 1% osmium tetroxide for 1 h at room temperature after a 2 h wash by 0.1 M phosphate buffer. After the cells were briefly washed with distilled water, routine dehydration was performed. The plastic embedded mount was prepared as described previously [10]. Ultrathin sections (100 nm) were cut on a Leica Ultracut microtome (Leica Microsystems,Wetzlar, Germany), collected on 200 mesh copper grids, stained with uranium and lead salts, and viewed on a Zeiss 910 transmission electron microscope (Zeiss Inc., Oberkochen, Germany).

### Quantitative real-time reverse transcriptase-PCR analysis

Total RNA was extracted using TRIzol (Invitrogen) according to the manufacturer’s instructions Trizol was joined according to 10 cm^2^/ml proportion. About 1 µg total RNA from each sample was reverse-transcribed into cDNA with the RevertAid™ First Strand cDNA Synthesis Kit.(Fermentans, Foster City, CA) in a total volume of 20 μl and stored at −20 °C. Real-time quantitative PCR was used to determine the optineurin, brain-derived neurotrophic factor (*Bdnf*), neurotrophin-3 (*Ntf3*), synaptosomal-associated protein 25 (*Snap25*) transcripts using a sequence-detection system (GeneAmp 5700; Applied Biosystems, Inc. [ABI], Foster City, CA). PCR reactions were performed in a 50 µl reaction mixture containing 25 µl master PCR mix (SYBR Green PCR Master Mix; ABI), 5 pM primer pairs, and 1 µl cDNA samples. Glyceraldehyde-3-phosphate dehydrogenase (*GAPDH*) was used at the same time as an internal control. Reactions were performed through the following conditions: 10 min at 95 °C for initial denaturation, 40 cycles of denaturing for 5 s at 95 °C, and annealing for 31 s at 60 °C. Experiments were repeated 3 times (n=3). All the primers used in the reactions are described in Table 2.

**Table 2:** Sequence of relative genes.

**Gene**	**Sequence (5′-3′)**
*GAPDH*	F: TGATTCTACCCACGGCAAGTT
	R: TGATGGGTTTCCCATTCATGA
Optineurin	F: GAAGGAGATGAGGAACAGCG
	R: CAGGAGT TG GCTCACAGTCA
*Bdnf*	F: AGGCACTGGAACTCGCAATG
	R: AAGGGCCCGAACATACGATT
*Ntf-3*	F: TGCAGAGCATAAGAGTCACC
	R: AAGTCAGTGCTCGGACGTAG
*Nefl*	F: CAGCGTGGGTAGCATAAC
	R: TGATTCACATTGCCGTAGA
*Snap-25*	F: TTTCCTTCCCTCCCTACC
	R: GGCATCGTTTGTTACCCT

### Protein isolation and western blot analysis

About 100 μl cell lysis buffer (60 mM of Tris, 2% sodium dodecyl sulfate (SDS), 100 mM of 2-mercaptoethanol, and 0.01% bromophenol blue) was added to each of the tissue samples. An equal amount of protein (10 μg) was loaded onto 10% sodium dodecyl sulfate-polyacrylamide gels, and electrophoresis was performed for 1 h. Following separation and transfer onto a polyvinylidene difluoride membrane (Invitrogen), the blots were incubated with 1:500 dilution optineurin or GAPDH primary antibody (Abcam, Cambridge, MA) at 4 °C overnight. Horseradish peroxidase (HRP)-conjugated antirabbit IgG secondary antibody was incubated at room temperature for 2 h at a 1:5,000 dilution. An Enhanced Chemiluminescence (ECL) kit (Amersham Bioscience, Piscataway, NJ) was used to detect blotting signals following the manufacturer’s instructions Mixed Reagents I and II according to 1:1,and then 10 times diluted. The amount of GAPDH in each sample was measured as an internal control. Gel-Pro Analyzer software (Media Cybernetics) was used to analyze the data.

The same western blot analysis protocol was used to detect Bdnf, Ntf3, Snap25, and Nefh protein expression in stable transfected RGC-5 cells. All of the antibodies were purchased from Abcam. Experiments were repeated 3 times per protein per treatment group (n=3).

### 3-(4,5)-dimethylthiazol (-z-y1)-3,5-di-phenyltetrazolium bromide assay

Approximately 200 μl of 3-(4,5)-dimethylthiazol (-z-y1)-3,5-di- phenyltetrazolium bromide (MTT; Sigma-Aldrich, St. Louis, MO) solution (5 mg/ml) was added into the RGC-5 and PC12 cells at 24 h, 48 h, and 72 h, respectively, after cell passage to 70% confluence. Cell plates were then wrapped with aluminum foil and incubated for 4 h at 37 °C. Cell plates were shaken for 15 min after the MTT was discarded, the precipitate was solubilized in 100% DMSO (Sigma) using 200 μl/well. Absorbance of each well was measured on a microplate reader (Wellwash MK2; Labsystems Dradon, Helsinki, Finland) at a wavelength of 490 nm. Experiments were repeated 3 times (n=3).

### Flow cytometry

Cell apoptosis was assessed with flow cytometry using an Annexin V FITC/PI staining kit (PharMingen, Becton Dickinson Co., San Diego, CA). After 48 h of transfection, we harvested cells, washed twice in phosphate buffer solution (PBS; sodium chloride NaCl 40.0 g, potassium chloride KCl 1.0 g, potassium dihydrogen phosphate anhydrous KH_2_PO_4_ 1.0g, disodium hydrogen phosphate anhydrous Na_2_HPO_4_ 4.6 g, distilled water to make up to 5 l; 4 °C), resuspended in binding buffer (10 mm HEPES/NaOH pH 7.4, 140 mm NaCl, 2.5 mM CaCl_2_), stained with fluorescein isothiocyanate-conjugated annexin V (Annexin V-FITC), mixed gently and incubated for 15 min at room temperature in the dark, and then washed with binding buffer and analyzed with flow cytometry (FACS Calibar; Becton-Dickinson) using CellQuest software (BD,San Jose, CA). Experiments were repeated 3 times (n=3).

### Microarray analysis

Agilent dual-channel cDNA microarray was used for these experiments. Total RNA was isolated from transfected and untransfected samples by TRIzol as described previously. Total RNA was further purified using a QIAGEN RNeasy® Mini Kit (Qiagen, Valencia, CA). A low RNA Input Linear Amplification Kit (Agilent Technologies, Wilmington, DE) was used for cDNA and cRNA synthesis. aaUTP (Ambion) was used to create amplified and labeled cRNA with T7 RNA polymerase, which also amplified the target material and incorporated Cy3- or Cy5-labeled CTP. cRNA from the stable transfected samples was amplified with the incorporation of Cy5-CTP, while cRNA from the control samples was labeled using Cy3-CTP and then purified with a QIAGEN RNeasy® Mini Kit. Cy3 and Cy5 labeled cRNA samples were maintained in fragmentation buffer at 60 °C for 30 min. Hybridization was completed for each sample with a whole separate Genome (4×44 K) microarray (Agilent Technologies) at 65 °C in a hybridization oven overnight. We then washed, stabilized, dried, and immediately scanned the hybridization slides using the Agilent G2565BA Microarray Scanner System (v 7.0). A single log2 ratio (SLR) was calculated from transfected and normal cells.

## Results

### Effects of optineurin siRNA on optineurin expression

**Figure 1:**
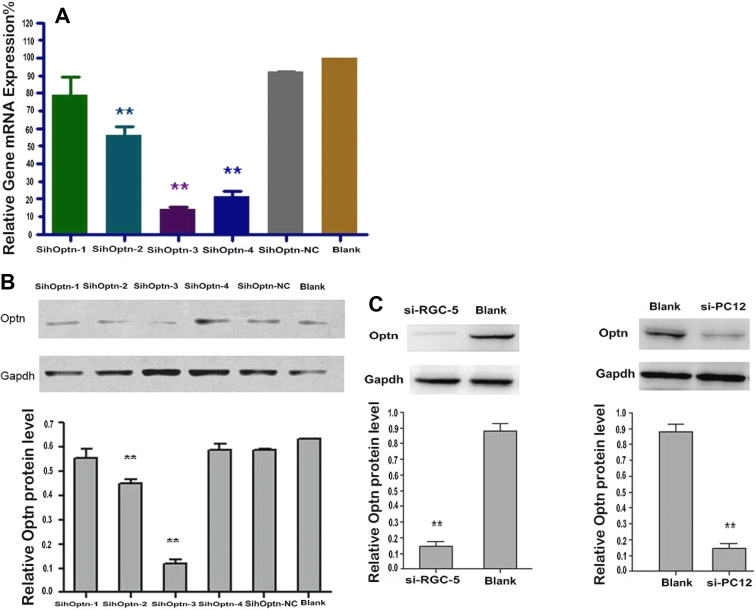
Effects of optineurin siRNA on optineurin expression in transfected cells. **A**: Quantitative real-time PCR was used to measure optineurin mRNA expression. Mock transfected cells were set to 100%. Optineurin siRNA no. 3 was the most effective in inhibiting optineurin mRNA expression and showed 14.34% of expression in Blank cells (p<0.01, n=3). *GAPDH* served as the endogenous control. **B**: western blot analysis was used to measure the amount of optineurin protein in sihoptineurin-1(1), sihoptineurin-2(2), sihoptineurin-3(3), sihoptineurin-4(4), sihoptineurin-NC(5) transient transfected and untransfected PC12 cells. The ratio of inhibition for sihoptineurin-3 was 83.1% (p<0.01, n=3). **C**: western blot analysis showed the knockdown of optineurin in sihoptineurin-3 stable transfected RGC-5 cells and stable transfected PC12 cells. Western blot showed that the inhibiting ratio of optineurin was 84.96±0.88% of Blank cells (p<0.01, n=3). GAPDH served as the loading control. (*p<0.05, **p<0.01, n=3).

Four optineurin siRNA–expressing plasmids (sihoptineurin-1, sihoptineurin-2, sihoptineurin-3, sihoptineurin-4) and one negative control (sihoptineurinNC) were transiently transfected into PC12 cells. The inhibition effectiveness of optineurin of the five siRNAs in PC12 cells was assayed with quantitative real-time PCR. As shown in Figure 1A, the transfected cells showed 78.99%, 56.34%, 14.34%, and 21.22% expression relative to the untransfected cells, respectively, of which sihoptineurin-2, −3, −4 showed a significant decrease compared to the control (p<0.01, n=3). The sihoptineurinNC transfected cells were not significantly different from the untransfected cells (92.02%, p>0.05, n=3), indicating that the transfection procedure did not affect optineurin expression. Western blot was used to validate the results of quantitative real-time PCR (Figure 1B). Sihoptineurin-3 was the most effective siRNA to inhibit optineurin protein expression and with 83.1% inhibition of expression versus untransfected cells (p<0.01, n=3). Therefore, we used sihoptineurin-3 to construct stably transfected cell lines. Using western blot (Figure 1C) and fluorescence microscopy to identify the cell lines, we constructed stable transfected sihoptineurin-3 RGC-5 and PC12 cell lines, which were used in the following experiments.

### Effects of optineurin siRNA on cell growth

**Figure 2:**
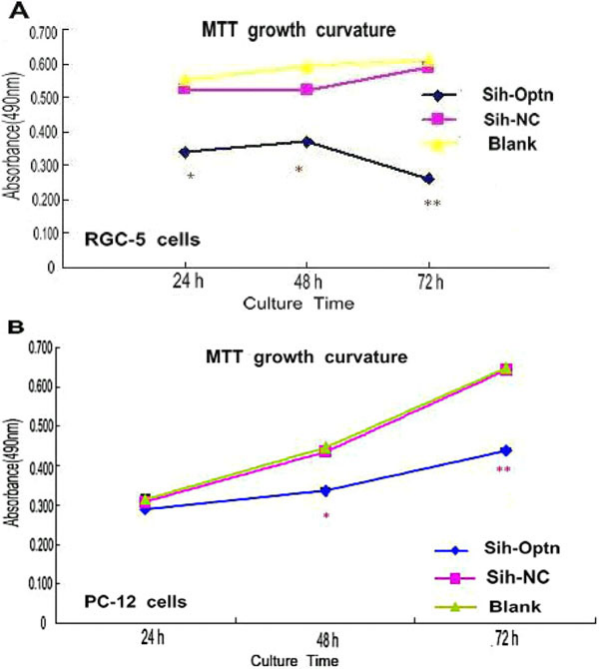
Effects of optineurin siRNA on RGC-5 and PC12 cell growth as measured by the MTT assay. *p<0.01 (versus sihoptineurin-NC and blank), **p<0.001 (versus sh-NC and blank) n=3. In the experimental groups, sihoptineurin-3 groups significantly inhibited cell growth compared to controls at 24 h, 48 h, and 72 h.

As shown in Figure 2 with the MTT assay, sihoptineurin-3 significantly inhibited cell growth in stable transfected RGC-5 and PC12 cells compared to controls. In the RGC-5 cells, sihoptineurin-3 significantly inhibited cell growth at 24 h (p<0.01, n=3), 48 h (p<0.01, n=3), and 72 h (p<0.001, n=3). In the stable transfected PC12 cells, cell growth was significantly inhibited at 48 h (p<0.01, n=3) and 72 h (p<0.001, n=3). There was no significant difference between the negative control (NC) and untransfected PC12 cells (Blank) after 3 days (p>0.05, n=3).

### Effects of optineurin siRNA on cell apoptosis

**Figure 3:**
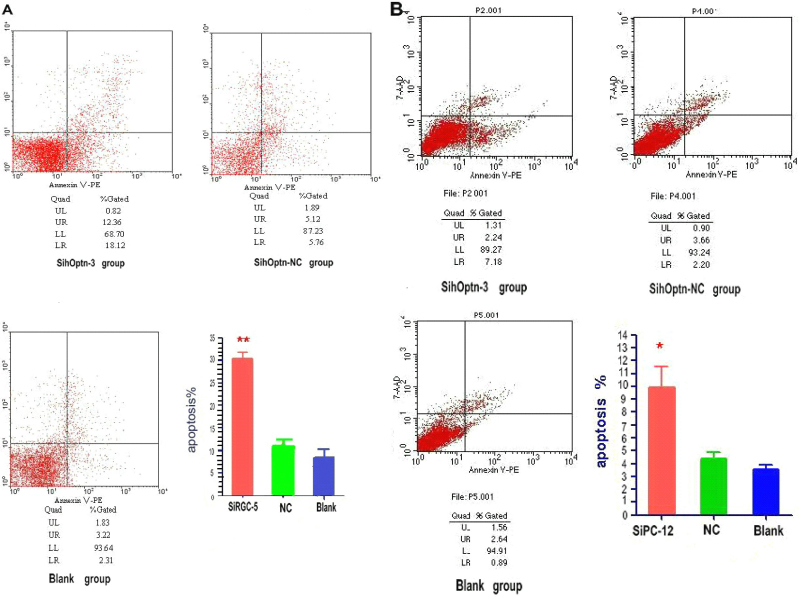
Effects of optineurin siRNA on apoptosis of stable transfected RGC-5 cells and PC12 cells as measured with flow cytometry. LR denotes early apoptosis, and UR is advanced stage apoptosis. (*p<0.05, **p<0.01), n=3. Apoptosis %=Gated (UR+LR)%. Apoptosis rate of experimental groups is obviously higher than that of sihoptineurin-NC and blank groups.

Flow cytometry was used to identify cell apoptosis in this study. Flow cytometry showed that cell apoptosis occurred in 30.41%±2.41% and 9.91±2.81% (SD) in sihoptineurin-3 stable transfected RGC-5 and PC12 cells, respectively, while 11.17%±2.77% and 8.09%±3.86% in RGC-5, and 4.36±0.87% and 3.55±0.55% in PC12 NC and untransfected control cells. There was a significant difference between the experimental groups and the controls (p<0.01 in RGC-5 and p<0.05 in PC-12, n=3; Figure 3).

**Figure 4:**
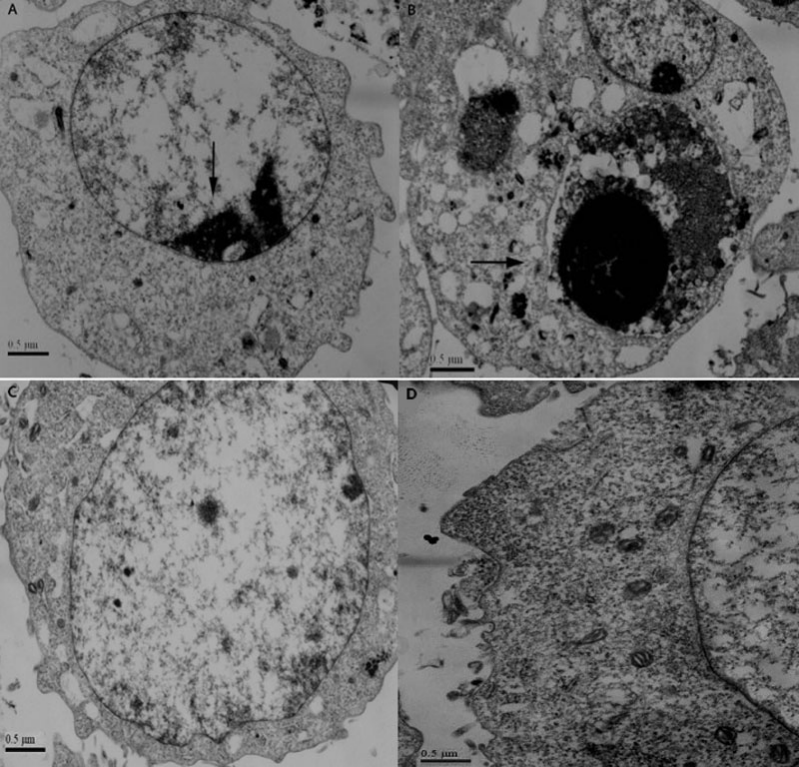
Electron microscopy showed apoptosis in RGC-5 cells after stable transfection of sihoptineurin-3. **A**: Nuclear heterochromatin margination, partial membrane dissolution, rough endoplasmic reticulum expansion, and mitochondrial reduction were observed in sihoptineurin-3 RGC-5 cells. **B**: Mitochondrial outer membrane damage, cell membrane partial dissolution, and apoptotic bodies were observed in sihoptineurin-3 RGC-5 cells. **C**, **D**: In sihoptineurin-NC RGC-5 and blank cells, we observed nuclear membrane integrity, with organelles enriched in cytoplasm.

Electron microscopy showed nuclear heterochromatin margination, partial membrane dissolution, rough endoplasmic reticulum expansion, mitochondrial reduction (Figure 4A), and mitochondrial outer membrane damage, cell membrane partial dissolution, and apoptotic bodies (Figure 4B) in the sihoptineurin-3 stable transfected RGC-5 cells. However, in the negative control and untransfected cells, we observed nuclear membrane integrity, with organelles enriched in cytoplasm (Figure 4C,D). These data indicated that optineurin is related to cell apoptosis.

### Microarray analysis

To investigate the molecular mechanisms underlying optineurin’s effects on cell growth and apoptosis, gene microarray analysis was applied to scan the differentially expressed genes in RGC-5 cells. One hundred twelve genes were identified as being upregulated by ≥1.0 SLR (fold change >2), or downregulated by <-1.0 SLR (fold change <-2) in RGC-5 stably transfected cells versus control. Genes with SLR>2.0 (fold change>4) or SLR<-2.0 (fold change<-4) were selected to do gene function catalog analysis and were found to be enriched in apoptosis, growth, and axonal transport (Appendix 1).

### Validation of differentially expressed genes in PC12 and RGCs cells

**Figure 5:**
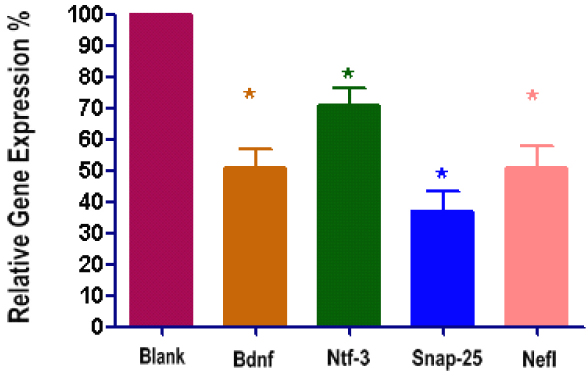
Validation of microarray data in stable transfected PC12 cells with quantitative real-time PCR. Bdnf was 57.52% of the expression in the Blank cells, Ntf3 was 62.42%, Snap25 was 36.40%, and Nefl was 55.66% (*p<0.05 versus control, n=3).

Four differentially expressed genes with SLR<-2.0 (fold change<-4) were further validated. In the PC12 stably transfected cells, quantitative real-time PCR showed that Ntf3 was 78.09% (p<0.05, n=3), Bdnf was 57.52% (p<0.05), Snap25 was 36.40% (p<0.05, n=3), and Nefl was 55.66% (p<0.05, n=3) of the expression relative to untransfected cells (Figure 5).

**Figure 6:**
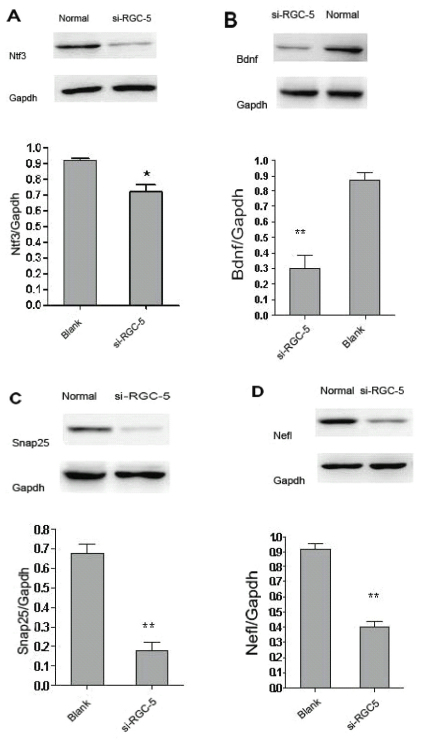
Western blot analysis examined the changes in selected protein expression in RGC-5 cells. Protein lysates from RGC-5 cells were probed with antibodies to Bdnf, Ntf3, Snap25, Nefl, and GAPDH. Optical densitometric analysis was performed to calculate the relative protein amounts of siRNA-treated cells compared to Blank cells. In the sihoptineurin-3 transfected RGC-5 cells, the expression of Bdnf was 2.06-fold, Ntf3 was 1.28-fold, Snap25 was 3.119-fold, and Nefl was 2.16-fold lower than that of Blank cells, respectively (p<0.05; *p<0.05, **p<0.01) n=3.

In the stable transfected RGC-5 cells, western blot analysis demonstrated that the expression of Bdnf was 2.06 fold, Ntf3 was 1.28 fold, Snap25 was 3.119 fold, and Nefl was 2.16 fold lower than that of normal cells (p<0.05, n=3; Figure 6).

## Discussion

After optineurin was identified in 1998 [11], Rezaie et al. [5] found that the incidence of POAG resulting from optineurin gene mutations was higher than the control group. Recent studies [12,13] revealed that optineurin genetic mutation and single nucleotide polymorphisms (SNPs) were associated with open angle glaucoma. However, the mechanisms have not been elucidated. To determine the comprehensive molecular mechanisms and signal transduction pathways of optineurin, Weisschuh et al. [9] used RNA interference to silence optineurin in HeLa cells, and, using microarray technology, found a series of differentially expressed genes. However, retinal ganglion cells are target cells of glaucoma, and optineurin is mainly located in the optic nerve [5,14]. Therefore, we chose RGC-5 and PC12 cells as our research targets and used siRNA to knock down the expression of optineurin in this study. RGC-5 cells are different from normal RGCs in electrophysiology and were more appropriate as neuronal retinal precursors [15], but are similar in terms of molecular and genetic changes during apoptosis and responses to neuroprotective agents [16-19]. Thus, RGC-5 cells are widely used as a model of RGC. PC12 was also applied in this study, because it is widely used to study neuronal development and function as a tissue culture model. We constructed four optineurin siRNA plasmids and transiently transfected them into PC12 cells. Quantitative real-time PCR and western blot demonstrated that all of the four siRNAs significantly decreased optineurin expression in PC12 cells. We selected the most effective one, sihoptineurin-3, to establish RGC-5 and PC12 stable transfected cells. The origination of the RGC-5 cell line has been controversial. RGC-5 cells were found to be of mouse [15] by sequencing a region of the nuclear Thy1 gene and the d-loop and tRNA(Phe) gene in mitochondrial DNA. However, several other studies [20-22] have found RGC-5 cells to be of rat origin. We therefore designed the optineurin-3 siRNA according to the rat optineurin cDNA sequence and found a significant effect of the siRNA on the expression of optineurin, as demonstrated by Q-PCR and western blot. Thus, the significant effect of optineurin-3 siRNA makes the origin of RGC-5 no longer an issue in this study because it already allowed us to investigate the consequences of inhibiting optineurin expression in the cell line.

In the other experiments, the MTT assay and flow cytometry showed that optineurin knockdown significantly inhibited cell growth and promoted apoptosis. There have been many controversies regarding whether optineurin promotes or inhibits cell apoptosis. Word et al. [23] speculated that optineurin was an important component of the TNF-alpha signaling pathway and activated apoptosis. Kroeber et al. [24] showed that overexpression of wild-type optineurin had no significant effects on lens epithelial cell apoptosis induced by TGF-beta1 in mice. Evans and Hollenberg [25] speculated that overexpression of optineurin might not be a damaging effect on the trabecular meshwork cells, but a protected gene. Takahisa Koga [26] found that apoptotic activity increased by force-expressing wild-type optineurin into RGC-5 cells. BumChan Park et al. [21] demonstrated that overexpressed wild type optineurin resulted in impairment of the Tf uptake in RPE and RGC-5 cells, which could induce apoptosis. Nevertheless, E50K cells induced more dramatic effects than the wild-type optineurin. Our results suggest that optineurin mutations may lead to increases in apoptosis and decreases in cell growth in retinal ganglion cells that may result in POAG. Differences in these results are due to the different types of cells and different conditions of experiments. Perhaps, optineurin plays different roles in different stages of apoptosis. Further studies are needed.

To further determine the molecular mechanisms underlying the effects of optineurin on cell apoptosis, we used microarray technology. We found many signaling pathways were involved, such as TNF-alpha/nuclear factor-kappaB [7,27] and Ifn [28], as previously reported. In our study, 112 differentially expressed genes were identified in RGC-5 cells, of which Bdnf, Nefl, Ntf3 and Snap25 were validated in PC12 cells with quantitative real-time PCR and in RGC-5 with western blot analysis. These results were not obtained in optineurin knockdown HeLa cells [9] due to the different cell types and stable transfected cells that we used for microarray analysis. For the first time, we have revealed that optineurin may regulate cell growth and apoptosis and transport of neurotransmitters through Bdnf, Ntf3, Nefl, and Snap25.

Snap25 and Nefl usually produce fast and slow transport of the axon [29,30], and Bdnf and Ntf3 are neuroprotective factors and could impact cellular proliferation and apoptosis [31,32]. Bdnf is a neuroprotectant following optic nerve injury [33] and can support the survival of RGCs in vivo [34-36]. Ntf3, which is a subset of neural crest and placode-derived neurons, plays a role in increasing survival and leading to neurite outgrowth. Ntf3 regulates the proliferation of cultured neural crest progenitor cells grown in a serum-free defined medium [37]. In addition, Ntf3 also acts as a survival factor for differentiated neurons in the retina and promotes neuronal differentiation [38]. Both Bdnf and Ntf3 might play roles in the development of the rat and chick retina [39,40]. Recent studies confirmed that Bdnf and Ntf3 could be used to treat RGC injury [36,41,42], since they could promote the survival of injured cells. In our research, we found that optineurin knockdown significantly downregulated Bdnf and Ntf3 expression that was probably related to the survival and apoptosis of retinal ganglia cells. This indicates that reduced expression of neuroprotective factors, such as Bdnf and Ntf3, may be one of the reasons that optineurin mutations lead to POAG.

Snap25 is located in the presynaptic plasma and plays an important role in the synaptic vesicle membrane docking and fusion pathway [43]. Brownlees et al. demonstrated that Nefl proteins affect axonal transport of neurofilaments and neurofilament assembly in cultured mammalian cells and neurons [29]. Snap25 and Nefl are usually implemented as producers of fast and slow transport [30,44]. In this study, we found that knockdown of optineurin in RGC-5 downregulated the expression of Snap25 and Nefl. The result suggested that optineurin might affect the general rates of slow and fast axonal transport in vitro and that optineurin is an essential component for axonal transport. Optineurin might affect the neurotransmitter and neurotrophic factor expression and transport in RGC apoptosis. We speculate that optineurin could be a protective gene for RGC injury, and that optineurin plays an important role in the axonal transport process.

In the future, further in vitro and in vivo studies are required to show that optineurin causes glaucoma via regulating Bdnf, Ntf3, Snap25, and Nef1.

## Appendix 1.

List of genes related to apoptosis and growth. To access the data, click or select the words “Appendix 1.” This will initiate the download of a compressed (pdf) archive that contains the file.
